# Surface-Related Features Responsible for Cytotoxic Behavior of MXenes Layered Materials Predicted with Machine Learning Approach

**DOI:** 10.3390/ma13143083

**Published:** 2020-07-10

**Authors:** Maciej E. Marchwiany, Magdalena Birowska, Mariusz Popielski, Jacek A. Majewski, Agnieszka M. Jastrzębska

**Affiliations:** 1 Interdisciplinary Centre for Mathematical and Computational Modelling (ICM), University of Warsaw, Pawińskiego 5a, 02-106 Warsaw, Poland; m.marchwiany@icm.edu.pl; 2 Faculty of Physics, University of Warsaw, Pasteura 5, 00-092 Warsaw, Poland; Mariusz.Popielski@fuw.edu.pl (M.P.); Jacek.Majewski@fuw.edu.pl (J.A.M.); 3 Faculty of Materials Science and Engineering, Warsaw University of Technology, Wołoska 141, 02-507 Warsaw, Poland; agnieszka.jastrzebska@pw.edu.pl

**Keywords:** machine learning, MXenes, cytotoxicity, van der Waals layered materials

## Abstract

To speed up the implementation of the two-dimensional materials in the development of potential biomedical applications, the toxicological aspects toward human health need to be addressed. Due to time-consuming and expensive analysis, only part of the continuously expanding family of 2D materials can be tested in vitro. The machine learning methods can be used—by extracting new insights from available biological data sets, and provide further guidance for experimental studies. This study identifies the most relevant highly surface-specific features that might be responsible for cytotoxic behavior of 2D materials, especially MXenes. In particular, two factors, namely, the presence of transition metal oxides and lithium atoms on the surface, are identified as cytotoxicity-generating features. The developed machine learning model succeeds in predicting toxicity for other 2D MXenes, previously not tested in vitro, and hence, is able to complement the existing knowledge coming from in vitro studies. Thus, we claim that it might be one of the solutions for reducing the number of toxicological studies needed, and allows for minimizing failures in future biological applications.

## 1. Introduction

Experimental development of two-dimensional (2D) materials is booming and the industrial applications are not only envisioned but also are becoming a reality. On the other hand, safety verification by systematic and in-depth studies pose a great challenge. The first stage of research into the safety of individual materials are the in vitro studies on mammalian cells. If material is nontoxic, they are moving forward into more advanced studies. In this regard, toxicity appears when the viability of tested cells decreases from 100% to below 70%. This means reduction over 30% which is significantly below the natural 20% variance of survival rate assumed for the living organisms.

One of the most perspective 2D materials that can be utilized in technological applications are the MXenes. During last years their development has risen exponentially. MXenes are defined as early transition metal carbides, nitrides, and carbonitrides. They have received much attention as their unique 2D crystal structure can be easily tuned to produce dramatic improvement in material properties [1]. Therefore, it is not surprising that they have been successfully applied in many fields of materials science and technology [2]. The term ‘MXene’ reflects the unique 2D structure of the material in which the formula M_n+1_X_n_T_z_ perfectly matches the arrangement of its layered features. In this formula—M is early transition metal, X is carbon and/or nitrogen, n = 1, 2, 3, or 4, whereas T_z_ corresponds to functional groups terminating the surface (e.g., -OH, = -O, -F) [3]. The family of MXenes has expanded rapidly since the discovery of their first representative—the Ti_3_C_2_T_z_ phase in 2011 by Naguib et al. [4]. Note, that the first package of MXenes included only several phases with 19+ successfully synthesized in subsequent years [5]. Since that time, ten years have passed and now researchers are able to predict new MXene phases theoretically [6], and new phases have been successfully synthesized (see Reference [7]).

In the case of MXenes cytotoxicity, the first studies concerned in vitro testing of multilayered Ti_3_C_2_T_z_ MXene and showed a potential threat related to the generation of reactive oxygen species (ROS) [8]. Further studies showed differences in toxicological effects in view of MXenes stoichiometry (i.e., Ti_3_C_2_T_z_ or Ti_2_CT_z_) [9]. Moreover, the importance of flake thickness was highlighted by us not only in the case of material stability but also potential toxicity [10]. In addition, cytotoxicity was also examined in respect to the number of layers constituting MXene compounds [11].

In this regard, we have come to the moment that innovative solutions are needed to extract the most promising representatives of MXenes with the highest potential for application and the lowest cytotoxicological threats. It becomes obvious that it is impossible to carry out screening investigations for all MXenes phases in reasonable time and manageable costs. The most time- and cost-consuming analyses are undoubtedly the biological studies, which are also inevitable to push through MXenes applications in industry. What is more, many certification procedures involve verification of the safety of market products containing the claimed nanomaterials. Accordingly, there is a strong demand for theoretical solutions that could overcome the problem of so many complicated analyses.

One such solutions might be the machine learning (ML) procedure. Machine learning has so far proved its applicability for cytotoxicity studies of large number of various chemicals (see Reference [12]) as well as recent predictions of synthesis of various MXenes compounds [13]. In addition, it can be also used to effectively analyse complex surface science data [14]. Thus, we assume that it might be one of the solutions for reducing the number of toxicological studies needed, and allows for minimizing failures in future biological applications. Machine learning studies concerning toxicity of drug, molecules have been carried out previously, by using deep learning and XGBoost [15] approaches, and using Atomic Fingerprints [16,17,18,19,20,21]. However, to the best of our knowledge, there is a lack of toxicological research based on ML methods concerning layered materials, in particular, 2D structures. Thus, we present the first approach of predicting the cytotoxicity of experimentally synthesized MXene compounds.

The aim of this study is to provide the prediction of the potential cytotoxic behavior of MXenes materials based on ML model with some elemental information provided from experiments. We first determine the biological and physico-chemical features that describe each 2D material in relation to the tested cytotoxicity. On this basis, we determine the possible descriptors for predicting the cytotoxic behavior of MXenes compounds. In particular, the surface characteristics, morphology, and structure have been taken into account as inputs for our theoretical model. Next, the Random Forest [22] approach has been applied to identify the most important features, that might have an impact on MXenes cytotoxicity. Then, we apply Principle Component Analysis (PCA) [23] as feature engineering to improve our model. We use the key features to train machine learning models. The models are checked by a 10-fold cross-validation scheme, with their performance measured by accuracy score, data set contains experimentally measured samples provided from our own research studies, as well as from screening experiments. Then, we use this model to predict the potential cytotoxic behavior of 19 experimentally examined MXene compounds not tested in vitro. It is crucial to note, that this study relies on small size of dataset, thus, we include variety of external methods to validate our predictions.

Note, that the theoretical methodology developed here can be further applied to other types of 2D materials, which exhibit diverse surface characteristics. The detailed information about the experimental data and elemental features used in this study are presented in Appendices Appendix A and Appendix B, respectively.

## 2. Materials and Methods

In order to determine a good quality ML model, it is crucial to determine potential descriptors that can characterize the cytotoxic behavior and identify ML algorithms well suited for a given dataset.

### 2.1. The Choice of Descriptors

Descriptors, which are representative of the compounds properties play a crucial role in ML, thus, we decided to describe in details the rationale behind the particular choice of the descriptors used in our studies.

At first, we choose only the MXenes compounds tested in vitro which are well experimentally characterised and appear cytotoxic or not. In this regard, the particular MXene exhibits cytotoxicity if it causes the reduction of viability of tested cells in vitro below 70% within the concentration range up to 250 $\frac{mg}{L}$. In addition, the ROS level above 120% at a concentration of 250 $\frac{mg}{L}$ is also an indicative of the presence of cytotoxicity. The rest of MXenes appears as non-cytotoxic. Furthermore, we gather and sort the information from material synthesis methodology as well as results from characterisation. This includes the chemical composition of MXene (M, X), surface modification with external compounds, lateral size, thickness, etching agent, delaminating agent, elements and oxides (M*_x_*O*_y_*) present on the surface which are studied by the X-ray photoelectron spectroscopy (XPS). Based on these considerations, we aim to select particular experimental descriptors listed in Table A2.

Based on the previous experiments [9] that linked the cytotoxic behavior of MXenes with the stoichiometry of the structures and thickness of the flakes, the data concerning the geometry information (lattice and atomic structures) about the known 2D MXenes compounds have been taken into account. The theoretical descriptors have been build on the basis of the theoretical dataset, namely atomic types and positions using Atom-Centered Symmetry Functions (ACSF) [24]. The theoretical descriptors are collected in the Table A3.

Note, that the choice of the descriptors which represent the cytotoxic behavior is also limited to the experimental data available in literature. Therefore, the prediction might change whenever more experimental data are provided.

### 2.2. Datasets

The toxicological in vitro data for 2D MXenes is taken from recently published high-throughput screening experiments, therefore, it is reliable and convenient for comparison. We have decided to test independently three datatsets in order to answer following questions:

1. What kind of datasets and descriptors: theoretical, experimental or combined ones are sufficient to build a good quality ML model?
2. Could the cytotoxic behavior be predicted based on purely theoretical descriptors (type of atoms, stoichiometry, etc.), and hence, does it mean that no experimental data need to be provided to predict the cytotoxic behavior?
3. Does inclusion of the geometrical descriptors in the ML model improve qualitatively predictions of the cytotoxicity?

Three datatsets are listed below. For each of the datasets the ML models have been built separately and discussed in details in the corresponding subsections of Results.

- Dataset I (experimental set)—the experimental data have been selected based on detailed description of the experiment, as well as detailed information about the structure, surface modification, in-depth characterization of morphology, and the cytotoxic effect of the MXenes compound on the cells tested in vitro. Those information have been collected from the literature listed in Table A1 and presented in Appendix A. It consists of 71 records and elemental features (descriptors) listed in Table A2.
- Dataset II (theoretical set)—data taken from the two-dimensional database [25] concerning the geometry information about the known 2D MXenes compounds (61 records). The elemental features are collected in Table A3.
- Dataset III (combined set)—dataset consists of both Dataset I and Dataset II. The number of the records and elemental features are combined from those two datasets correspondingly.

It is worth mentioning, that the first two datasets overlapped partially, namely, the geometry of each of the compounds in the first set is known. In addition, we do not know anything about the class of function describing cytotoxicity, so it is not possible to point out the class of algorithms that should be used. Thus, only the size and the type of the variables in the dataset determine our choice of ML algorithms. Detailed analysis of the applicability of machine learning algorithms can be found elsewhere [26]. Below, we present briefly the ML algorithms used in the present study:

- Logistic regression [27] with regularization L_1_ and L_2_ (regLOG-L_1_, regLOG-L_2_). This approach allows avoiding over-learning a model even for a large number of variables. The algorithm removes unimportant features for the model.
- Random Forest (RF) [22] is commonly used for a small dataset, and must be used with care regarding over-learning. It allows for selecting the most important features.
- Support Vector Machine (SVM) [28] uses only part of the dataset, thus, it can be easily applied to a small size of dataset. The key point of prediction in the SVM algorithm is the choice of kernel. In this study, we have tested the commonly used kernels such as—linear, rbf, and sigmoid, denoted by us regSVM-lin, regSVM-rbf, regSVN-sig, respectively, throughout this paper.
- Extreme Random Tree (ERT) [29] is an extension of a Random Forest algorithm, and is known to be computationally faster than RF. Both ERT and RF are known to work well for any dataset.

Parametric models such as linear regression are used to help us understand a phenomenon by determining the functional dependences. In the case of non-parametric models such as Random Forest, the crucial issue is to identify the importance of features, and thus, it allows us to understand the studied phenomenon. Note, that other commonly used ML methods such as Kernel Ridge Regression (KKR) [30] or Neural Networks (NN) [31], are well suited for large datasets, thus, are not applicable in our case.

In addition, our datasets face a commonly known issue, namely, the class imbalance problem. Significantly, this problem is widely reported for the toxicity of many other materials, where the size of the positive data (toxic samples) is considerably smaller than the negative data (non-toxic samples) (see Reference [32]). To solve this problem, we make use of various data-balancing algorithms such as—Weight classifier (Weight), and generating synthetic samples (SMOTE). The other commonly used algorithms such as oversample minority class or undersample majority class are not applicable in the case of MXenes materials, due to the small number of toxic records for which proper statistics cannot be built.

We have used the Python programming language (version 3.6.4) with the scikit-learn [33] and XRT [34] libraries for data analysis and machine learning. The Pandas [35] library was adopted in order to read and process the data, whereas the NumPy package (version 1.16.1) [36] was used to construct the features.

## 3. Results

In this study, we make use of state-of-the-art machine learning (ML) methods to identify the cytotoxicity of experimentally synthesized as well as deeply characterized 2D MXenes.

This section is divided into three subsections related to the prediction for the three datasets: experimental set, theoretical set and combined one, as it was mentioned in previous section. Then, the models are simplified, by selecting the most important features based on Random Forest algorithm or by the construction of new features from the given ones by the use of the Principle Component Analysis (PCA). The models are tested by 10-fold cross-validation, with the performance measured by class balanced accuracy score of correct predictions [37]. The accuracy score metric is defined in the range of [0, 1].

### 3.1. Dataset I—Experimental Set

Our theoretical investigations (see Table 1) reveal that the accuracy score for balanced data shows a good level of precision, greater than 0.72 (except for regSVM-sig) for all of the algorithms employed in this paper. Moreover, note that data balancing techniques improve the results approximately by a few percent (see the results for balanced versus unbalanced data collected in Table 1). The largest values are obtained in the case of the SVM with rbf kernel (regSVN-rbf) for Weight and SMOTE techniques, and are equal to 0.92 and 0.93 respectively. The high accuracy obtained in the case of regRF and regSVM-rbf manifest the non-linearity of the studied problem and the need for using non-parametric models. Unfortunately, this has a negative effect on understanding of the results and the underlying phenomenon. It is worth noting, that most of the variables used in this study are categorical variables described by one-hot-encoded or labeling methods. By use of the SMOTE data balancing algorithm, the results do not include pure features but contain mixed ones, which results in losing the physical interpretation of the outcomes. Thus, we have decided to use the Weight data balancing algorithms further in this study.

In order to understand the cytotoxicity issue and its dependency on selected descriptors, feature selection and feature engineering techniques are applied further in this study. The crucial premise of feature selection is that the data contains some variables that are either redundant or irrelevant, and can thus be removed without much loss of information. Feature selection and feature engineering techniques are methodological tools that allow simplification of the model, and hence, they can facilitate the interpretation of the studied phenomenon. In order to select the most important features, the feature importance score is obtained from a random forest analysis. Feature importance shows how much weight the model assigns to the given descriptor during predictions, and thus, gives insight into which variables are crucial for predicting the cytotoxicity of MXenes materials. For comparison purposes, we also use feature engineering from PCA.

**Figure 1:**
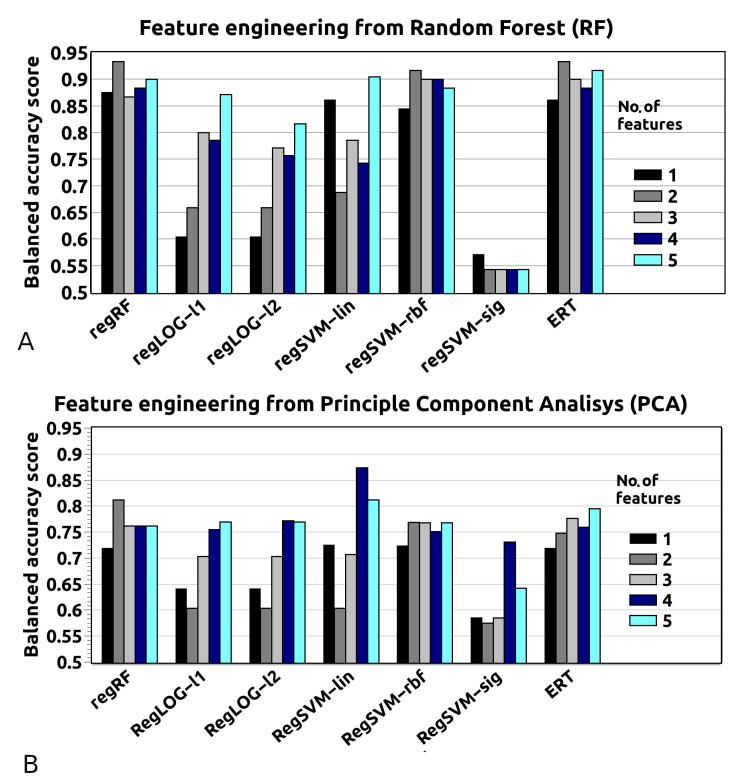
Feature engineering for dataset I obtained for two methods: (**A**) Random Forest (RF) and (**B**) Principle Component Analysis (PCA). Both methods are methodological tools that allow simplification of the model.

The results reveal that three features are already sufficient for a good level of prediction accuracy, for all employed algorithms except regSVM-sig (see Figure 1).

Moreover, all the models based on PCA show lower accuracy than RF, which means that there is a low correlation between the features. The PCA approach is based on correlation between the features, while feature importance from RF selects the most important, original variables. PCA does not improve the results, thus, the original variables selected by RF have been chosen.

**Figure 2:**
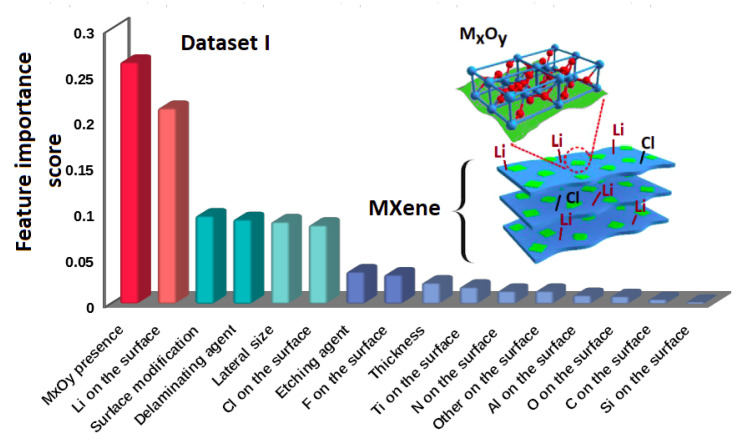
Ranking of feature importance obtained from RF. The most important are two descriptors: (i) the presence of M*_x_*O*_y_*, and (ii) Li on the surface, whereas the next four: surface modification, delaminating agent, lateral size, and Cl on the surface are equally important with smaller weights than the previous two. All feature labels are described in Table A2.

In addition, the feature importance score shows that there are two crucial features, four equally important features, and the rest seem to be unimportant from the RF analysis (see Figure 2). The most important features are the presence of the M*_x_*O*_y_* and the Li atoms on the MXenes surfaces.

Note that the results presented here show that experimental data and descriptors are sufficient to build a good quality ML model for cytotoxicity predictions in MXenes materials.

### 3.2. Dataset II—Experimental Set

Here, the ML model is built based on theoretical data. We have tested the dataset with structural information of the compounds included, namely position and type of atoms. There are many methods available for building descriptors such as Atom-Centered Symmetry Functions (ACSF) [24], Coulomb Matrix [38], or Ewald Sum Matrix [39], which convert the atomic positions into variables that can be used in machine learning. We have used the Weighted Atom-Centered Symmetry Functions (wACSF) [40] as descriptors, in order to substantially decrease the number of variables. The parameters of this model have been adopted from Reference [24]. Note that only 10% records of dataset have been labeled as toxic or non-toxic. In order to effectively elucidate the information contained in this dataset, clustering technique has been applied. Therefore, we have used unsupervised ML techniques. All these methods do not allow us to correctly distinguish toxic and non-toxic records in two separate clusters. In other words, the results reveal that the clustering technique cannot be viewed as a mechanism for toxicity prediction of MXenes.

All the results presented in this subsection reveal that taking into account geometrical descriptors from theoretical dataset of MXenes materials are not sufficient to build a ML model for cytotoxicity prediction of MXenes compounds. However, this theoretical set can be combined with experimental set, to determine whether such enlarged database improves model predictions, which is a subject of study in the next subsection.

### 3.3. Dataset III—Combined Experimental and Theoretical Sets

Our study reveals that including the information related to the geometry of the compounds (see Table 2) does not improve the results, and gives a similar level of accuracy score of correct predictions as obtained for dataset I (see Table 1), for all of the methods employed here.

**Figure 3:**
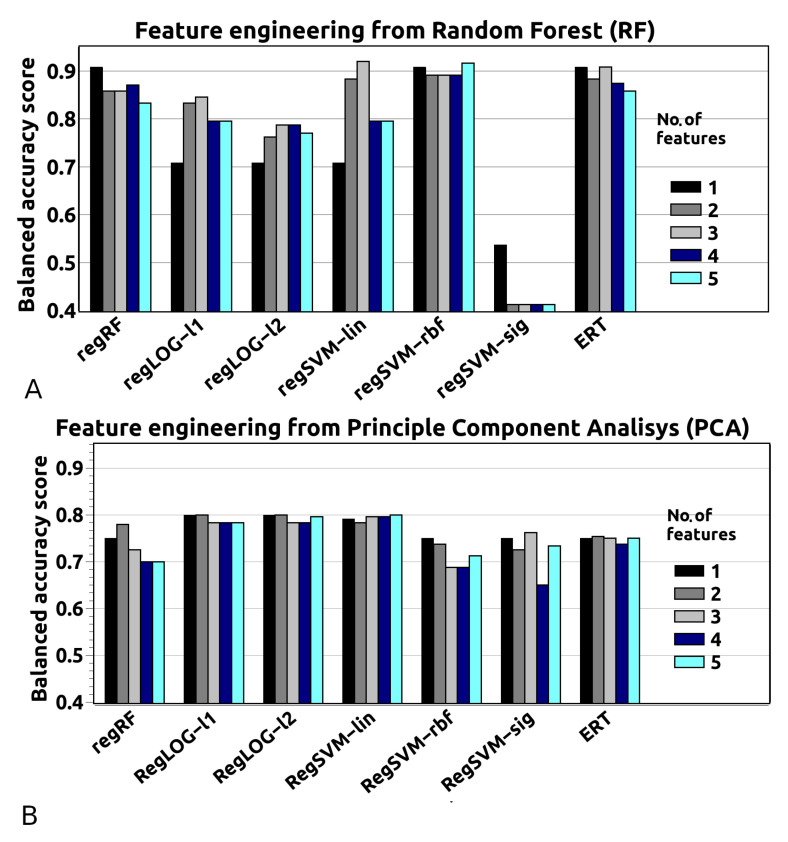
Feature engineering for dataset III obtained for two methods: (**A**) Random Forest (RF) and (**B**) Principle Component Analysis (PCA). Both methods are methodological tools that allow simplification of the model.

**Figure 4:**
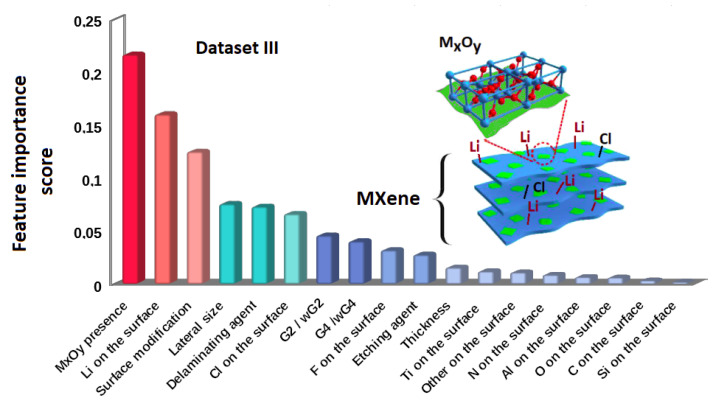
Ranking of feature importance for dataset III. The most important are the first three descriptors, namely M*_x_*O*_y_*, Li on the surface and surface modifications, respectively. All feature labels are described in Table A2 and Table A3.

Then, we have built models that include from one to five features by use of the Random Forest algorithm and PCA method, similarly to the approach for dataset I. The analysis demonstrates that two features are sufficient to describe the toxicity of MXenes compounds with high accuracy of predictions (see Figure 3). From the feature importance ranking, we find out that the topmost descriptors are the presence of M*_x_*O*_y_*, Li on the surface of MXenes, and surface modification with external compounds (see Figure 4). Note, that the order of the six top important features is the same as in the case for dataset I.

### 3.4. Discussion of Datasets

Here, we present the discussion concerning the results of employed datatsets in response to the following questions (previously stated in Section 2.2):

1. What kind of datasets and descriptors: theoretical, experimental or combined ones are sufficient to build a good quality ML model?
  Our results indicate that experimental data are sufficient to build an effective model with high accuracy score of correct cytotoxicity predictions based on 10-fold cross-validation scheme.
2. Could the cytotoxic behavior be predicted based on purely theoretical descriptors (type of atoms, stoichiometry etc.) and hence, does it mean that no experimental data need to be provided to predict the cytotoxic behavior?
  Our results reveal that taking into account only geometrical descriptors from theoretical dataset of MXenes materials are not sufficient to build a ML model for cytotoxicity predictions. Thus, the experimental information such as material synthesis methodology and characterisations play a crucial role in building ML model concerning cytotoxic behavior of MXenes compounds.
3. Does inclusion of the geometrical descriptors in the ML model improve qualitatively predictions of the cytotoxicity?
  The inclusion of geometrical features of MXenes do not qualitatively change the ML results based on experimental dataset.

## 4. Model Predictions

After successful verification of the ML model based on experimental dataset, we are able to predict toxicity for 2D MXenes not tested in vitro. To do so, we have searched the available literature covering the MXenes compounds for which all the elemental features listed in Table A2 have been provided, but with no in-vitro studies carried out. Despite the fact that there are around a hundred phases synthesized so far, we have only found 19 MXenes compounds, for which comprehensive data on the material are available (see Table 3).

The ML models predict two of 2D MXenes can exhibit cytotoxic properties with a high probability of prediction equal to 0.9, while the rest of them are predicted to be non-toxic (see Table 3). It is worth mentioning that for the non-toxic ones, no presence of M*_x_*O*_y_* on the surface has been reported. The presence of M*_x_*O*_y_* is the key toxicity-generating feature obtained from our studies.

Our results demonstrate that our ML model is able to complement the existing knowledge coming from in vitro studies. However, note that this prediction has to be viewed with some caution, knowing that traditional k-fold cross-validation is highly optimistic when evaluating machine learning models, due to the fact that materials datasets are rarely uniformly distributed.

## 5. Discussion

Our results indicate that knowledge of the surface and its modification might be crucial issue concerning the toxicity of the studied layered 2D materials, whereas geometrical descriptors may have little impact on the outcomes. These results are in the line with recent experimental findings concerning the presence of Ti*_x_*O*_y_* on the surface [8,58,59], and the biological knowledge of cytotoxicity mechanisms [60], as well as physical and chemical intuition. It should be stressed that this conclusion is much more definitive than we expected at the beginning of our studies. The reason is that the chemical diversity and inhomogeneity of MXenes are already widely known and pose a major challenge in such complex analysis. The second corresponding aspect is the divided surface characteristics. The primary strictly depends on the starting materials (MAX phases). The latter is undoubtedly far more problematic, if it comes into interactions with highly sensitive systems such as biological ones. Basically, the chemical composition of the surface of MXenes, almost certainly, is closely related to the type of ‘M’ element and a resulting chemical composition of the M*_x_*O*_y_* passivation layer that occurs as a result of M reaction with oxygen and/or water [61]. In fact, every surface of the MXene exposed to the air can naturally react with oxygen because the freshly exposed metallic surface is energetically unsaturated and possesses high reactivity. This can happen immediately after the delamination process (but certainly must also depend on the MXene stability). What’s more, the freshly exposed surface of the MXene also acquires bonding with products of chemical reactions that occur during acidic etching of the ‘A’ element from the MAX phase. As can be seen, the aforementioned surface-related features influence at first the material itself, but finally, may result in different biological effects such as the appearance or lack of cytotoxicity.

From this regard, MXenes are the most interesting, because their surface is highly unstable and susceptible to oxidation in the ambient conditions [52]. It has been shown that the surface effects highly affect the living cells and it is closely linked to the oxidation process and decomposition to toxic oxides. In such a case, mechanisms of cytotoxic action refer to cell cycle, DNA synthesis, and cellular membrane integrity [62]. From this perspective, surface chemistry of MXenes should be managed to avoid further toxic effects using redirecting into more safe surface compositions [59]. This is indeed a right direction when the surface modification cannot be used. In other cases, polyanionic salts can be effectively used for MXene flakes edges capping which results in highly decreased decomposition and stability [50]. In other approach, natural antioxidants are good in diminishing surface oxidation [49]. In addition, approaches that involve surface modification with bio-organic moieties, specifically designed for cytotoxicity mitigation, are also highly recommended [62,63].

## 6. Conclusions

Here, we present the first theoretical study concerning the toxicological aspects of 2D MXene materials by employing various machine learning models. Our work demonstrate that the most important features potentially responsible for the toxicological properties are related to the presence of transition metal oxides M*_x_*O*_y_* and Lithium atoms on the surface, as well as surface modification with external compounds. Our detailed analysis reveals, that the crucial issue is what happens on the surface, while the structural information of the systems might have minimal impact on cytotoxicological aspects of MXenes materials.

Our ML model successfully complement existing experimental studies, for which no cytotoxicological measurements have been carried out. In particular, we have predicted the cytotoxicity of 19 MXenes compounds, for which two of them are predicted to be cytotoxic with 0.9 probability. The rest of the compounds are predicted to be non-toxic and can be potentially applied in many technological areas [2,64,65,66,67,68].

Moreover, our results show that the cytotoxic prediction of MXenes can be examined on the materials that are well experimentally characterised in terms of surface chemistry and the presence of oxides on the MXenes surface. Thus, we claim that it might be one of the solutions for reducing the number of toxicological studies needed, and allows for minimizing failures in future biological applications.

In addition, the theoretical research methodology based on ML models developed here can be further applied to other types of 2D materials exhibiting complex structure and diverse surface characteristics, such as for example, novel 2D transition metal borides, so called MBenes [69] as well as van der Waals heterostructures [70,71]. We expect that the predictions presented here will facilitate the experimental efforts by providing the information that might accelerate time consuming and expensive cytotoxical experimental studies, by reducing the large number of compounds, and hence, speeding up potential future applications.

## Figures and Tables

**Table 1:** The accuracy score of correct predictions are obtained for unbalanced and balanced data, for the various algorithms employed in this paper. The most accurate results are obtained for balanced data by use of the Support Vector Machine with rbf kernel (regSVM-rbf).

		Balanced Data	
ML Algorithms	Unbalanced Data	Weight	SMOTE
regRF	0.747	0.826	0.833
regLOG-l1	0.700	0.776	0.783
regLOG-l2	0.720	0.798	0.783
regSVM-lin	0.867	0.725	0.808
regSVM-rbf	0.662	0.917	0.933
regSVM-sigmoid	0.555	0.543	0.4042
ERT	0.722	0.776	0.750

**Table 2:** The metric of accuracy score of correct predictions is obtained, for various of algorithms employed in this paper.

ML Algorithms	Model Selection: Weight
regRF	0.845
regLOG-l1	0.845
regLOG-l2	0.727
regSVM-lin	0.781
regSVM-rbf	0.876
regSVM-sigmoid	0.545
ERT	0.793

**Table 3:** Predicted probability of cytotoxicity score of various MXenes compounds. For each of the compounds different chemical treatment, as well as chemical composition on the surface have been reported. Thus, different probability scores have been obtained. All data taken from high-throughput screening experiments (see last column). The abbreviation Us. denotes Ultrasounds, whereas the the third and fourth columns denote the presence of the $M_{x}O_{y}$ and Li atoms on the surface. Note, that two descriptors etching agent and delaminating agent are denoted here as the synthesis procedure.

MXene [Ref.]:	Probability	M*_x_*O*_y_*	Li Atoms	Synthesis Procedure	Surface Mod.
Ti_3_C_2_ [41]	0.18	No	Yes	LiF/HCl; Us.	No
Ti_3_C_2_ [42]	0.05	No	No	HF	Au
Ti_3_C_2_ [43]	0.18	No	Yes	LiF/HCl; Us.	No
Ti_3_C_2_ [44]	0.10	No	No	HF; Us.	No
Ti_3_C_2_ [45]	0.10	No	No	HF; Us.	No
Ti_3_C_2_ [46]	0.06	No	Yes	LiF/HCl	APTES + CEA
Ti_3_C_2_ [47]	0.07	No	Yes	LiF/HCl; Us.	DNA, Pt, Pd
Ti_3_C_2_ [48]	0.05	No	No	HF; Us.	Ag
Ti_3_C_2_ [49]	0.07	No	Yes	LiF/HCl	L-ACC
Ti_3_C_2_ [50]	0.07	No	Yes	LiF/HCl; Us.	PAS
Ti_3_C_2_ [51]	0.87	Yes	No	LiF/HCl	No
Ti_3_C_2_ [52]	0.88	Yes	No	LiF/HCl	No
V_2_C [50]	0.05	No	Yes	LiF/HCl; Us.	PAS
V_2_C [53]	0.05	No	No	NaF/HCl	No
Nb_2_C [54]	0.04	No	No	NaF/HCl	No
Nb_2_C [55]	0.04	No	No	HF; 60^∘^	No
Ti_2_N [10]	0.05	No	No	KF/HCl; Us.	No
Mo_1.33_C [56]	0.04	No	No	HF; TBAOH	No
Ti_4_N_3_ [57]	0.03	No	Yes	KF/LiF/NaF, TBAOH	No

## Abbreviations

The following abbreviations are used in this manuscript:

SP	Soybean phospholipid
PVP	polyvinylpyrrolidone
HA	hyaluronic acid
PEI	polyethylene imine
PEG	polyethylene glycol
PVA	polyvinyl alcohol
NMP	N-methyl-2-pyrrolidone
TMAOH	tetramethylammonium hydroxide
TBAOH	tetrabuthylammonium hydroxide
TPAOH	tetrapropylammonium hydroxide
DMF	dimethylformamide
DMSO	dimethyl sulfoxide
CTAC	cetanecyltrimethylammonium chloride
c(RGDyC)	cyclic arginine-glycine-aspartic pentapeptide
PLL	poly-L-lysine
APTES	(3-aminopropyl) triethoxysilane
CEA	carcinoembryonic antigen
L-ACC	L-ascorbic acid
PAS	polyanionic salts

## Appendix A. Detailed Information about the Experimental Data Used in the Machine Learning Models

Here we present the list of the high-throughput experimental data that we used to build the database (see Table A1).

**Table A1:** The types of delaminated 2D MXenes compounds used in this study. The information about the particular compound are presented in details in the following references.

MXenes Compound:	
Ti_3_C_2_	References [8,41,42,43,44,45,46,47,48,49,50,51,52,72,73,74,75,76,77,78,79,80,81]
Ta_4_C_3_	References [82,83,84]
Nb_2_C	References [54,55,85,86]
Ti_2_C	Reference [67]
Mo_2_C	References [87]
Mo_1.33_C	Reference [56]
Nb_4_C_3_	References [62]
V_2_C	References [50,53]
Ti_2_N	Reference [10]
Ti_4_N_3_	Reference [57]

## Appendix B. Detailed Information about the Elemental Features Used in the Machine Learning Models

Here we present the detailed information about the elemental features taken into account in the machine learning predictions, listed in Table A2 and Table A3 respectively.

**Table A2:** Detailed description of the elemental features used in ML scheme and applied for the dataset I and dataset III.

Detailed Description of the Feature	
Surface modification	PVP, SP, MnO*_x_*+SP, HA, Fe*_x_*O*_y_*+SP, PEI, PEG, DNA+Pt+Pd, Ag, L-ACC, PAS
with external compounds	CTAC+PEG+SiO_2_+c(RGDyC)+SiO_2_, Au+Fe_3_O_4_, Au+PEG, PLL, APTES+CEA, PVA, Au
Lateral size	from few to hundredths of nm, from few to hundredths of μm
Thickness	from few to tens of nm
Etching agent	HF, LiF+HCl, LiF+HCl+AlCl_3_, NaF+HCl, KF+HCl, KF+LiF+NaF,
Delaminating agent	Ultrasounds, DMF+high pressure+high Temp., high pressure+high Temp., TBAOH, TBAOH+ultrasounds, TPAOH, TMAOH, DMSO+ultrasounds, no additional treatment
Carbon (C) on a surface	1-Yes, 0-No
Oxygen (O) on a surface	1-Yes, 0-No
Fluor (F) on a surface	1-Yes, 0-No
Aluminium (Al) on a surface	1-Yes, 0-No
Titanium (Ti) on a surface	1-Yes, 0-No
Nitrogen (N) on a surface	1-Yes, 0-No
Chloride (Cl) on a surface	1-Yes, 0-No
Silicon (Si) on a surface	1-Yes, 0-No
Lithium (Li) on a surface	1-Yes, 0-No
Other on a surface	1-Yes, 0-No
presence of M*_x_*O*_y_*	1-Yes, 0-No
Toxic	1-Yes, 0-No

**Table A3:** Detailed description of the elemental features used in ML scheme for the dataset II and constituting the part of the dataset III.

$G^{2}\setminus wG^{2}$	see Reference [24]; cutoff R_C_ = 6, shift R_s_=(0⋯N), number of the functions N = 10, $\eta =(1,N^{\frac{R_{s}}{N}})$
$G^{4}\setminus wG^{4}$	see Reference [24]; cutoff R_C_ = 6, shift R_s_=(0⋯N), number of the functions N = 10, $\lambda =\left\{-1,1\right\}$, $\zeta =\left\{1,2,4,16\right\}$
